# Robot-assisted repair of ureteral stricture

**DOI:** 10.1007/s11701-024-01993-9

**Published:** 2024-09-28

**Authors:** Mu-Yang Xu, Zheng-yao Song, Chao-Zhao Liang

**Affiliations:** 1 Department of Urology, The First Affiliated Hospital of Anhui Medical University, Anhui Medical University, No. 218 Jixi Road, Hefei, 230022 Anhui People’s Republic of China; 2 Institute of Urology, Anhui Medical University, Hefei, Anhui People’s Republic of China; 3 Anhui Province Key Laboratory of Urological and Andrological Diseases Research and Medical Transformation, Anhui Medical University, Hefei, Anhui People’s Republic of China

**Keywords:** Robotic urinary tract reconstruction, Ureteral stricture, Diversion surgery, Robot-assisted buccal mucosa graft (BMG) ureter reconstruction, Robot-assisted urinary tract reimplantation

## Abstract

As robot-assisted laparoscopic techniques continue to advance, becoming increasingly complex and refined, there has been significant progress in the minimally invasive treatment of ureteral strictures. This abstract aims to provide an overview and description of various surgical techniques that utilize robots for repairing ureteral strictures. We have summarized the progression of these surgical methods and highlighted the latest advancements in the procedures. When compared to open surgery, robot-assisted reconstruction techniques demonstrate superior functional outcomes, fewer postoperative complications, and a faster recovery in the treatment of ureteral strictures. This abstract aims to provide an overview and description of various surgical techniques utilizing robots to repair ureteral strictures. Robotic ureteral stricture correction has emerged as a valuable therapeutic option, particularly when endoscopic procedures are not feasible. Compared to traditional open surgery, robotic methods exhibit superior therapeutic effectiveness, fewer postoperative complications, and accelerated recovery. Reconstructive procedures such as reimplantation, psoas hitch, Boari flap, ureter-to-ureter anastomosis, appendix graft, buccal mucosa graft (BMG), ileal transplantation, or kidney autotransplantation can be performed depending on the extent and location of the stricture. Robotic surgical techniques also offer advantages, such as an expanded field of vision and the incorporation of supplementary technologies such as FireflyTM, indocyanine green (ICG), and near-infrared fluorescence (NIRF) imaging. However, further long-term, multicenter investigations are necessary to validate the positive findings reported in existing case series. Compared with open surgery, robot-assisted reconstruction techniques yield superior functional outcomes, fewer postoperative complications, and accelerated recovery for the treatment of ureteral strictures.

## Introduction

The causes of ureteral stricture may be iatrogenic, traumatic, congenital, immunological, infection-related, inflammatory, or caused by stones. Among these, previous endoscopic procedures, such as endoscopic treatment of urologic stones and ureteroscopy, are the most common iatrogenic causes [1, 2]. Endoscopic intervention has a positive prognosis for patients with untreated, nonischemic, benign ureteral strictures that are smaller than 2 cm and affect functioning renal units [3]. However, endoscopic treatment is often suboptimal for strictures longer than 2 cm [4]. Surgical options for strictures longer than 2 cm in the distal, middle, or proximal segments of the urinary tract include ureter-to-ureter anastomosis (UU), ureteral reimplantation [5], psoas hitches [6], Boari flaps [7], appendiceal flaps [8], buccal mucosa graft (BMG) ureteroplasty [9], ileal replacement [10] and renal autotransplantation [11].

Prior to surgery, it is important to conduct a comprehensive evaluation that includes renal function tests and CT scans to identify the exact position and duration of the stricture accurately. Within the surgical setting, intravenous or retrograde pyelography, as well as flexible ureteroscopy with fluoroscopy, is available as options for precisely identifying the location of the stricture. The positioning of the trocar for distal ureter strictures is consistent with that of previous pelvic operations, such as radical cystectomy or robot-assisted prostatectomy. For mid- and proximal ureter strictures, it is preferable to use a 60-degree lateral decubitus position. This position helps reduce the need to adjust the operating table to extend the area excessively [12]. Minimally invasive reconstruction of the urinary tract must adhere to the established principles of open surgery, including delicate handling of the urinary tract to avoid ureter damage, optimizing the protection of blood supply to the ureter, ensuring tension-free connections, and strengthening the reconstruction with additional flaps on the peritoneum or perinephric fat. This approach aids in providing additional blood circulation and serves to avoid leakage. The incorporation of near-infrared fluorescence (NIRF) with indocyanine green (ICG) is a widely adopted innovation that greatly streamlines surgical procedures. The use of ureteral injection of ICG followed by visualization under NIRF allows quick and accurate identification of the ureter, facilitating precise localization of both the proximal and distal segments. Consequently, this approach improves the clarity of the boundaries of RUR ureteral strictures [13]. Following the administration of ICG, tissues with sufficient blood circulation exhibit discernible green fluorescence, but scar tissue does not display any fluorescence. ICG can be delivered via the opening of the renal pelvis [14], or it can be injected retrogradely into the urinary tract using a ureteral catheter. Both procedures can be employed concurrently. This approach facilitates accurate localization and comprehension of the anatomical features of the stricture during surgery, assisting in the identification of the most effective rebuilding approach.

With the development of science and technology, robot technology has been widely used in the surgical treatment of ureteral stricture. Since it has outstanding results and many benefits, such as improved ergonomics, enhanced visual magnification, flexible joint movement, elimination of hand tremors, and the ability to incorporate other imaging techniques such as near-infrared fluorescence (NIRF) imaging, Indocyanine green (ICG) and FireflyTM imaging, patients can expect reduced surgical complications, accelerated recovery, and a good functional prognosis. This article reviews the various categories and the latest progress in robotic correction of ureteral stricture, which provides a valuable perspective for the surgical treatment of ureteral stricture.

## Materials and methods

A comprehensive literature search was carried out using PubMed. The search included the following as individual terms and in combination: “ureteral reconstruction”, “buccal graft”, “appendiceal interposition”, “ileal ureter”, “autotransplantation” “Boari flap”, “Psoas hitch”. No date criteria were utilized. All articles were considered for the review including case reports, single-center studies, review articles, and meta-analyses. 87 articles were assessed and analyzed for patient characteristics and procedural outcomes. This review article presents an interpretation of results from the articles assessed. A standardized meta-analysis was not conducted; however, this review article aims to provide a synthesis of ureteral reconstruction techniques and the advantages of robot-assisted over traditional laparoscopy to guide management decisions.

### Robotic ureterotomy

Ureter-to-ureter anastomosis, also known as end-to-end anastomosis, is a highly effective method used to repair ureteral strictures in the proximal and mid-segments. These strictures are typically 2 to 3 cm long. Unlike ureteral reimplantation or a Boari flap, this method preserves the original structure of the bladder and prevents urine from flowing back into the ureter [15]. In 1922, Nezhat and colleagues performed the first laparoscopic ureter-to-ureter anastomosis (LU), which resulted in functional outcomes similar to those of open surgery but with improved esthetics [16]. The prognosis was favorable [17]. However, the widespread use of LU is limited by the need for extensive laparoscopic expertise and a restricted operating area, making it difficult to achieve anatomical precision and precise suturing. In 2006, Yee performed the first recorded instance of robot-assisted ureter-to-ureter anastomosis (RAUU) [18]. According to certain operators, RAUU is considered the most effective surgical method for removing short ureteral strictures (less than 3 cm) that do not respond to endoscopic therapy.

When the patient is placed in a lateral posture, it is most effective to start the anatomical procedure from the unaffected section of the ureter and progressively advance toward the constricted area. ICG and NIRF are used to identify and outline strictures. For example, a 6-Fr ureteral catheter is inserted prior to surgery, and during the procedure, 10 mL of diluted ICG is injected above and below the narrowing using the catheter. According to the authors, the disappearance or decreased fluorescence of the affected ureter clearly indicates the upper and lower boundaries of the ureteral stricture [19]. It is crucial to preserve the integrity of the outer layer and its blood supply. Incisions were made at both ends of the ureter, and the ends were sutured together using continuous single 4/0 or 5/0 sutures to create a surgical connection. Subsequently, a ureteral stent was placed and removed at the outpatient clinic 6 weeks after the operation. Repair posterior to the peritoneum reduces the risk of fistula development.

Hemal et al. reported on a series of seven patients in which retrograde antegrade ureteral stenting (RAUU) was used to treat strictures in the proximal ureter. The average operating room time (ORT) was 110 min, ranging from 85 to 140 min. The average estimated blood loss (EBL) was 50 mL, ranging from less than 50 to 75 mL. The average length of stay (LOS) was 3 days, ranging from 2 to 6 days. The mean duration of follow-up was 28 months, during which there were no complications during or after surgery and no recurrences of ureteral stricture [12]. Sun et al. conducted a study comparing 65 robotic procedures with 61 laparoscopic surgeries in adult patients. The results showed that the robotic group had shorter operation times, suture times, and lengths of hospital stay than did the conventional group. In addition, the robotic group exhibited lower levels of inflammation [20]. Maria Buffi conducted a survey involving 17 patients who underwent RAUU treatment. The median operative duration was 150 min, ranging from 100 to 420 min. The postoperative complication rate was 17.6%, with 3 patients experiencing complications. Specifically, 2 patients had Clavien–Dindo class I complications, and 1 patient had a grade II complication. The percentage of recurrence-free patients 2 years after RAUU treatment was 94.1% (ranging from 65.0% to 99.1%) [21].

### Robotic ureteral reimplantation (with or without psoas hitch)

Robot-assisted ureter repair, also known as ureter bladder anastomosis, is suitable for distal ureter strictures that are within 5 cm of the entrance of the urethra. The benefit of pelvic muscle ligation lies in its ability to maintain the integrity of the ureter epithelium and protect the functionality of the unaffected contralateral ureter. In addition, this method reduces the risks associated with persistent urinary tract infections and electrolyte imbalances. Inaugural laparoscopic ureter bladder anastomosis was conducted in 1994 by Reddy and colleagues [22]. Yohannes and colleagues later performed a procedure in 2003 in which robots were used to assist in the restoration of the urethra for patients with distal ureteral strictures [23]. Subsequent studies have outlined other methods [24].

The positioning of the trocar is similar to that of robot-assisted radical prostatectomy (RALP). The urethra was cut at the constricted front end and divided at the back edge. A bladder flap is utilized to aid in direct reimplantation of the urethra by suturing the mucosa of the bladder and urethra together. The posterior plate was secured with continuous 4/0 sutures, followed by the placement of a double-J stent and finalization of the anterior anastomosis. The bladder catheter was kept in place for 5–7 days.

The rectus fascia sling operation is used to repair gaps ranging from 6 to 10 cm in length. In this procedure, the Retzius space is enlarged, and the anterior branch of the bladder on the opposite side is surgically removed to realign the bladder. The lateral bladder wall is stitched using 3–4 nonabsorbable 2/0 sutures that travel through the rectus muscle tendons in a longitudinal manner to prevent harm to the inguinal nerve. Alternatively, the bladder can be secured to the peritoneal sidewall to achieve a tension-free connection. The bifurcated urethra is inserted into a submucosal passageway, with the length of the passageway adjusted to accommodate greater ureter expansion. A double-J stent was then placed, and the bladder was sealed [25–27].

Patil et al. conducted a study on robot-assisted distal ureteral reconstructions for benign diseases in adults and reported no complications during or after surgery, demonstrating the viability of robotic technology for treating distal ureteral strictures [28]. Casale and colleagues conducted a study on the largest collection of robot-assisted bladder extravesical reimplantation cases, effectively treating strictures without observing any significant complications [29]. However, comprehensive research comparing open versus robot-assisted ureter repair is lacking.

Kozinn et al. performed a comparative analysis that included 24 open reconstructions and 10 cases of robot-assisted reconstruction. The results showed a reduced duration of hospitalization and potentially decreased blood loss in the group that received assistance from robots. No stricture recurrence occurred in either group during the 2-year follow-up period [30].

Elsamra examined a total of 125 cases of ureter reconstruction, comprising 20 cases of open surgery, 20 cases of laparoscopic surgery, and 85 cases of robot-assisted surgery. The present study revealed no disparities in terms of stricture recurrence rates or surgery duration across the three groups. However, patients who underwent minimally invasive procedures had shorter hospital stays and less blood loss (P < 0.02) [31].

Schomburg et al. conducted a comparative study between 20 instances of open surgery and 20 instances of robot-assisted ureter repair and found no substantial disparities in estimated blood loss (EBL) or length of hospital stay (LOH), with comparable occurrences of surgical complications. The group that used robot assistance had a longer duration of surgery, but the amount of opioids used post-surgery was significantly lower than that used in the group that did not utilize robots (robo: 0.14 mg/kg, open: 0.25 mg/kg, *p* < 0.021). The severity of complications in the open group was frequently milder than that in the robot-assisted ureter reconstruction (RALUR) group [32].

Schiavina et al. compared 16 cases of laparoscopic ureter reconstruction with 12 cases of robot-assisted ureter reconstruction, including rectus fascia sling procedures. No significant disparities were observed in the incidence of stricture recurrence between the two groups. However, the laparoscopic group experienced a slightly short duration of hospitalization (*P* < 0.006). The robot-assisted group showed a decrease in blood loss that was statistically significant (*P* < 0.004) [33]. Future research investigating cost-effectiveness will be crucial for identifying the most effective way to treat this condition.

The Boari flap is an alternative method used to treat abnormalities in the middle part of the urethra when a narrow removal results in major abnormalities due to a long damaged segment, making it impractical to connect the urethra and bladder. The Boari flap was initially documented in 1947 [34]. In 2001, Fugita et al. introduced the laparoscopic Boari flap operation for treating lengthy ureteral blockage. No patients underwent successful surgery without complications, and no stricture recurred during an average follow-up period of 11 months [35]. Yohannes and colleagues utilized the Boari flap to reimplant distal strictures in 2003, marking the initial implementation of robotic ureteral stricture repair technology [23]. The robotic method uses a conventional trocar arrangement frequently employed in pelvic urological surgery, mobilizing the bladder on both sides before tying and separating the urachus and the pedicle of the opposite bladder. The superior vesical artery is used as a reference point for locating the base of the flap, which is then moved diagonally over the front wall of the bladder to the desired length. The incised ureter was repaired to the posterior wall using absorbable stitches. Double-J stents were implanted, and the front part was reshaped into a tube and sealed with uninterrupted sutures. The bladder is finally secured to the psoas muscle using nonabsorbable stitches. Indocyanine green (ICG) can be injected intravenously at any stage of the anastomosis procedure to verify vascular supply adequacy [5].

Due to the infrequency of this clinical issue and the complexity of the surgical procedure, there is a lack of extensive expertise. Castillo et al. reported a success rate of 96.6% in a group of 30 patients with positive short-term and long-term results [36]. In 2016, Stolzenburg et al. conducted a study recording the largest series of patient comprising 11 patients with benign ureteral strictures who underwent robot-assisted laparoscopic (RAL) surgery combined with Boari flap reimplantation. The success rate was comparable, with a complication rate of 9% [37, 38].

### Robot-assisted appendiceal covering flap

Utilizing the appendix as a replacement for tissue for treating proximal and mid-ureter lesions 2–6 cm in length is a favorable alternative, eliminating the need to employ other sections of the intestine, such as the ileum [39]. The initial case report dates back to 1912 and was produced by Melnikoff. Melnikoff employed the appendix in a tubular manner for this purpose [40]. The following authors have embraced this method and achieved favorable results. Both individual case reports from single institutions and case series from several institutions indicate that interventions involving ureteral appendiceal surgery and ureteroplasty with an appendiceal covering flap are both safe and feasible, with a success rate of 92%. Nevertheless, these operations also have problems, such as fistulas and recurring strictures [41]. It is mainly suitable for treating ureter strictures on the right side, but it can also be used to reconstruct ureter strictures on the left side.

In 2009, Reggio et al. reported the initial instance of laparoscopic appendiceal ureterostomy for the purpose of rebuilding ureter strictures. The study included an 8-month follow-up period [45]. This surgery involves cutting the appendix at its base while keeping its blood supply intact and then making it into a tube shape by making a lengthy incision on the side opposite to where the blood vessels are located. The stricture was detected, and excised, and its length was assessed using a ureter catheter. Afterwards, the appendix is stitched in a consistent and coordinated manner to the front side.

The results of this method show promise; however, the proof is limited due to the small number of documented cases. Duty et al. documented laparoscopic extravesical repair in six patients, for a success rate of 66% in four out of six patients [42]. A case series investigation conducted by Burns et al. showed that nine patients experienced positive long-term outcomes [43].

In their latest publication, Wang et al. presented their first robotic experience utilizing an augmented anastomotic repair technique, which achieved a 100% success rate at the 6-month follow-up [44]. Due to technological developments, this approach may soon become a compelling choice.

### Cheek mucosal graft urethroplasty

Cheek mucosal graft urethroplasty, also known as BMG urethroplasty, is a very efficient method specifically designed for treating ureter strictures longer than 3 cm that cannot be repaired through urethra-to-urethra connections. This technique is particularly advantageous for repairing the ureter because it reduces damage to the fragile blood supply of the ureter and makes it easier to create seamless connection without any stress. BMG ureteroplasty is especially beneficial for patients who have long ureteral strictures that cannot be treated with ureteroureterostomy and for those who have recurrent strictures after unsuccessful prior reconstruction. The utilization of cheek mucosa in the field of urology offers several benefits. It is conveniently reachable, has a low rate of illness or death [45–47], has characteristics resembling the lining of the urinary tract [48, 49], and is well suited for a damp setting [50].

In 1983, Naude and Somerville conducted initial studies on baboons to explore the use of buccal mucosa transplant ureteroplasty [51]. In 1999, Naude documented the first human series with six patients who were treated using three distinct procedures. In total, four patients underwent BMG ureteroplasty, which involved removing the damaged section of the ureter and repairing it using a BMG graft. A different patient underwent a surgical procedure called enhanced anastomosis BMG ureteroplasty, which involved removing the damaged section of the ureter, connecting the healthy part of the ureter, and repairing it using a BMG graft. A single patient underwent tubular BMG ureteral replacement. Throughout the 24-month observation period, there was no stricture recurrence. Previous studies have shown that the recurrence rates for buccal mucosa externalized or tubularized ureter repair range from 71 to 100% [52]. Kroepfl and colleagues conducted a case series in which they used open surgical surgery with BMG to treat long-segment ureter strictures. The success rate of this treatment was 71.4%, with a median follow-up of 18 months (ranging from 10 to 85 months). A single patient experienced repeated narrowing of the ureter 39 months after surgery and received temporary implantation of a stent in the renal ureter as a treatment. Another patient experienced recurring narrowing of the ureter 17 months after surgery and was treated by the insertion of a long-term renal ureteral stent [53].

In 2015, Zhao et al. introduced the concept of robot-assisted BGM ureteroplasty by demonstrating the approach in four patients with proximal ureteral strictures. The operation, intended for proximal ureter strictures with a median length of 4 cm (ranging from 1.5 to 6 cm), yielded positive results throughout the 15.5-month monitoring period. There were no instances of reoccurring conditions, and none of the patients required further surgical intervention. The BGM ureteroplasty procedure was performed with robot assistance, with the patient positioned on their side. The ureter was carefully identified and dissected during surgery. The use of fluoroscopy, ureteroscopy, intravenous injection, and near-infrared fluorescence imaging allowed accurate identification of the stricture either antegrade or retrograde ICG administration. A linear cut was made at the location of the narrowing in the urethra. The decision to use either buccal mucosa exteriorization or buccal mucosa-enhanced anastomosis was based on the scar’s length and severity, in addition to the support provided by the omentum or perinephric fat [54].

In 2017, Lee et al. published the most extensive collection of cases documented thus far, which included 12 patients who had robot-assisted BGM ureteral repair. Indocyanine green (ICG) was administered in a backward and/or forward direction into the inner lining of the ureter, and near-infrared fluorescence (NIRF) was used to visually detect ICG to pinpoint the beginning and end points of narrowing in the ureter. The strictures had a median length of 3 cm, with a range of 2–5 cm. The larger omentum was used to enclose the rebuilt ureters in all instances. A success rate of 83.3% (10 out of 12 patients) was reported [55]. In 2018, Zhao et al. conducted an inaugural multicenter study to evaluate the reproducibility of the approach. This study included 19 individuals from 3 distinct medical centers, with a median stricture length of 4 cm. The overall success rate reached a commendable 90% [47]. Lee, Zhao, and colleagues evaluated three robotic procedures specifically developed for treating moderate to long-segment (≥ 4 cm) strictures in the proximal urethra. The techniques mentioned are robot-assisted procedures for connecting the urethra to the urethra via downward renal fixation, connecting the urethra to the renal pelvis via downward renal fixation, and using a buccal mucosal graft for urethroplasty with the assistance of a robot. Based on a 24-month median follow-up, their data showed that 92.9% (13 out of 14 patients) of those who had RU-BMG had a successful outcome, with an interquartile range of 14–39 [9, 56, 57].

Ultimately, BMG urethroplasty emerges as a compelling option for treating strictures located in the proximal and mid-segments of the urethra. It can be regarded as a primary or secondary choice and may also demonstrate potential in the treatment of distal ureter strictures. However, additional multi-institutional research and longer term follow-up are necessary to confirm the complete array of benefits and drawbacks of these methods.

### Ileal replacement

Intestinal anastomosis is a viable alternative for replacing the ureter in patients with long-lasting ureteral strictures where endoscopic therapy or retrograde ureteroscopy is not suitable. It can also be considered salvage therapy after failed previous treatments and may be used in individuals with recurring kidney stone formation. However, this approach is not recommended for individuals with inflammatory bowel disease, radiation enteritis, neurogenic bladder, bladder outlet obstruction, or liver or kidney dysfunction [58, 59].

In 1906, Shoemaker was the first to use the ileum as a replacement for the ureter. This method gained popularity when Goodwin popularized it in 1959 [60]. Gill and colleagues performed the first laparoscopic surgery in the year 2000 using extracorporeal ileal anastomosis [61]. Wagner and colleagues conducted the inaugural machine-assisted ileal anastomosis for ureteral reconstruction surgery in 2008 [62]. In 2014, Brandao and colleagues reported the initial case of intracorporeal ureteral ileal replacement surgery. Regardless of the patient’s condition, the surgical procedure lasted for more than 6 h. However, the patients did not experience any significant issues, and there were no instances of the condition recurring [63]. Kocot conducted a comprehensive case series study to evaluate the long-term outcomes of 157 patients who underwent ileal ureteral insertion surgery; this is the largest study of its kind reported in the literature. The results indicated that 19.5% of the patients suffered from metabolic acidosis, while 6.3% had recurrent pyelonephritis. A significant proportion of patients, specifically 93.6%, experienced a reduction or maintenance of blood creatinine levels. In summary, remedial surgery was required in only 4.2% of patients [64].

The operation involved two main steps: excising the constricted portion of the ureter, followed by isolating a sufficiently long and well-supplied segment of the ileum. A stapler is used to restore the intestinal connection while placing the ileal ureter behind the peritoneum. Subsequent procedures include connecting the renal pelvis and the branch of the ileal bladder, as well as inserting a ureter stent to prevent leakage and maintain normal intestinal movement [65].

In 2016, Chopra and colleagues published a study on a group of 4 patients, with an average surgical duration of 450 min (ranging from 420 to 540 min) [66]. Stein et al. (2009) showed that laparoscopic ileal ureteral surgery has advantages over open surgery in terms of recovery time and length of hospital stay [67]. In 2019, Grosso et al. performed a surgical procedure called robot-assisted ileal ureteral replacement. The median duration of the operation was 270 min, with a range of 240 to 300 min. No problems, according to the Clavien–Dindo classification, were reported [68]. Soyster and colleagues conducted a retrospective study on a cohort of 160 patients who underwent ileal ureteral (IU) surgery. Researchers have shown that ileal ureteral repair is feasible, preserves renal function, and reduces long-term complications [69]. However, additional multi-institutional investigations are essential.

Robot-assisted techniques offer improved surgical visualization and accuracy but require time-consuming readjustment and repositioning. However, Ubrig et al. presented a robot-assisted method that allows the completion of the process without requiring the patient to be repositioned [70].

### Robot-assisted autologous kidney transplantation

Robot-assisted autologous kidney transplantation (RATx) is a viable therapeutic option for disorders such as lengthy ureteral or panureteral strictures, retroperitoneal fibrosis, loin pain-hematuria syndrome, and renal vascular problems (aneurysms, thrombosis, stenosis, and vascular damage). Although this approach produces positive functional results, it is associated with a rather high morbidity rate of 46% [71, 72]. An autologous transplantation procedure was initially conducted in 1963 using an open surgical technique involving a midline incision from the xiphoid process to the pubic symphysis. This surgery was performed to address serious damage in the proximal ureter [73]. Open autologous transplantation after laparoscopic kidney removal has been reported to be a viable and effective choice, with success rates varying from 68 to 90% [74].

Robot-assisted autologous renal transplantation (R-RATx) has demonstrated efficacy and achieved favorable outcomes. Gordon et al. documented the first total intracorporeal R-RATx, which required a total surgical duration of 425 min. The duration of warm ischemia was 2.3 min, while the duration of cold ischemia was 95.5 min. After the robotic removal of the donor kidney and cold perfusion, the kidney was placed in the iliac fossa, and the robot was deactivated. Afterwards, the patient was placed in a steep Trendelenburg posture, and the robot was positioned between the legs. Subsequently, the anastomosis between the iliac artery and ureter was prepared. Venous anastomosis required 17.3 min, while arterial anastomosis took 21.3 min. The duration required for the kidney to warm up (from the moment when cold perfusion stopped to the point where the kidney was released and reperfused) was 28.8 min. The ureterovesical anastomosis was successfully performed in a duration of 26.6 min. The amount of blood loss recorded was 50 ml [75]. Lee et al. made various alterations to R-RATx with the objective of diminishing kidney ischemia time. The modifications involved employing a Vicryl inner ring device to fasten the catheter and implementing cold HTK perfusion with saline. The surgical procedure lasted 390 min, during which warm ischemia time and cold ischemia time were measured at 4 and 48 min, respectively [76]. Sood et al. conducted the initial instance of robot-assisted R-RATx [77], whereas Araki et al. carried out the first instance outside of the United States [78]. Decaestecker et al. conducted the initial series investigation, comprising 7 patients, which involved the combination of hand-assisted and total intracorporeal approaches. The durations of surgery, warm ischemia, and cold ischemia were 370 min, 2 min, and 178 min, respectively [79].

R-RATx is deemed viable for treating intricate ureteral strictures in the future [84, 85]. Nevertheless, it is crucial to regard this approach as a viable alternative solely because of the possession of exceptionally skilled robotic surgeons and subsequent completion of transplantation-specific training.

### Reconstruction of the ureter after uretero-anastomotic stricture

The robot-assisted repair of ureteral stenosis remains applicable for cases where stenosis recurs following endoscopic or surgical interventions. Redo repair after ureteral anastomotic stenosis may become complicated due to the risk of significant periureteral fibrosis, changes in the dissecting plane, and further impairment of the fragile ureteral blood supply. Historically, open surgery has been commonly utilized for such cases. Current research indicates that robot-assisted surgery for recurrent ureteral stenosis post-reconstruction is equally effective as open surgery. Furthermore, robot-assisted technology demonstrates a reduction in blood loss and surgical time, thereby offering the advantages of minimally invasive surgery [80].

For the treatment of recurrent UPJO, options include pyeloplasty revision with a pelvis flap, cup incision of the ureter, and downward nephropexy (DN) or robotic-assisted ureteropelvic junction reconstruction (RU-BMG) [81]. For the treatment of recurrent proximal or mid-ureteral strictures, options include ureteroureterostomy, with or without DN or RU-BMG. In patients with localized fibrosis around the ureter and recurrent strictures in the proximal and mid-ureter (≤ 2 cm), ureteroureterostomy may be performed if feasible; otherwise, RU-BMG with a high-implant or augmented anastomosis is likely in most other cases [82]. For the treatment of recurrent distal ureteral strictures, we employ ureteroneocystostomy, ureteroureterostomy, ureteroureterostomy, or appendiceal interposition [83, 84].

Therefore, for patients with recurrent strictures following failed endoscopic or surgical treatments, robotic ureteral reconstruction (RUR) can be performed, with studies indicating favorable midterm outcomes [85]. While outcomes of using robotic reconstruction for re-stenosed uretero-anastomotic are promising, there is a lack of long-term follow-up data. In addition, the possibility of recurrent strictures despite robotic-assisted treatment of uretero-anastomotic stricture presents a challenge that needs to be addressed.

## Limitations of the literature

The existing body of research on robot-assisted ureteral repair techniques is primarily limited by the prevalence of small-scale studies conducted within a single institution. These studies also lack well-defined criteria for evaluating the effectiveness of the procedures. However, robot-assisted ureteral repair appears to be a viable and efficient alternative for treating this disease, yielding significantly more positive results and fewer complications than open procedures.

## Other patient considerations

Ureteral strictures present a significant challenge for surgeons, especially when they are of considerable length or unresponsive to endoscopic treatment and thus require surgical intervention. Robot-assisted surgery provides surgeons with several advantages, such as an extended visual range, enhanced accuracy, reduced invasiveness, lower complication rates, quicker recovery, favorable functional outcomes, and improved cosmetic results.

## Conclusion

Robot-assisted approaches for repairing ureteral strictures have emerged as a viable option, yielding positive functional outcomes and demonstrating lower complication rates than conventional open surgeries. However, extensive and long-term research studies are needed. Given the infrequent occurrence of this complex condition, there is currently a scarcity of scientific information available.

## Data Availability

No datasets were generated or analyzed during the current study.
